# Exploring the utility of Geometric Morphometrics to analyse prehistoric hand stencils

**DOI:** 10.1038/s41598-024-56889-3

**Published:** 2024-03-15

**Authors:** V. Fernández Navarro, R. M. Godinho, D. García Martínez, D. Garate Maidagan

**Affiliations:** 1 Instituto Internacional de Investigaciones Prehistóricas de Cantabria (IIIPC), Universidad de Cantabria, Gobierno de Cantabria, Santander, Avenida de los Castros s/n, 39005 Santander, Spain; 2 Interdisciplinary Center for Archaeology and Evolution of Human Behaviour (ICArHEB), Faculdade das Ciências Humanas e Sociais, University of Algarve, Faro, Portugal; 3 Physical Anthropology Unit, Department of Biodiversity, Ecology, and Evolution, Faculty of Biological Sciences, Complutense University of Madrid, Madrid, Spain

**Keywords:** Anthropology, Archaeology, Cultural evolution, Evolutionary developmental biology

## Abstract

Hand stencils are a remarkable graphic expression in Prehistoric rock art, dating back to 42 ka BP. Although these stencils provide direct impressions of the artists’ hands, the characterization of their biological profile (i.e., biological sex and age) is very challenging. Previous studies have attempted this analysis with traditional morphometrics (TM), whereas little research has been undertaken using Geometric Morphometrics (GM), a method widely used in other disciplines but only tentatively employed in rock art studies. However, the large variation in relative finger position in archaeological hands poses the question of whether these representations can be examined through GM, or, in contrast, if this creates an unmanageable error in the results. To address this issue, a 2D hand scans sample of 70 living individuals (F = 35; M = 35) has been collected in three standardized positions (n = 210) and digitized with 32 2D conventional landmarks. Results show that the intra-individual distance (mean Procrustes distance between Pos. 1–2 = 0.132; 2–3 = 0.191; 1–3 = 0.292) is larger than the inter-individual distance (mean in 1 = 0.122; 2 = 0.142; 3 = 0.165). Finally, it has been demonstrated that the relative finger positions, as well as the inclusion of all hand parts in the analysis, have an overshadowing effect on other variables potentially involved in the morphometric variability of the hand, such as biological sex.

## Introduction

Rock art is one of the earliest visual expressions in humankind, dating back (at least) to as early as 45.500 years BP, on the Indonesian island of Sulawesi^1^. The artistic corpus is varied, including the depiction of animals, hunting scenes, hand stencils, anthropomorphic figures and symbols. Altogether, such artistic expressions provide critical cultural, environmental, and subsistence insights into the past peoples that created them.

Hand stencils are a recurrent and ubiquitous rock art expression with the earliest examples found in the caves on Sulawesi with documented minimum U-Th dates of 37.2 ka BP for Lubang Jeriji Saléh^2^, and 32.29 ± 0.24/39.67 ± 0.32/32.60 ± 0.76 for Leang Balangajia 1, Leang Jarie and Leang Sampeang respectively^3^, among others. Further early examples are Castillo Cave (Spain) with a date of 37.63 ± 0.34 ka BP (minimum age)^4^ and Maltravieso (Spain) with 41.68 + 2.44/ − 2.29 ka cal BP and 70.08 + 3.82/ − 3.37 ka cal BP^5^.

In some cases, hand images are positive painted impressions of hands held directly against the cave surface. In other cases, pigment was blown over the hand and adhered to the supporting rock, thus resulting in a negative impression of the hand or ‘stencil’. In either case, hand images provide a proxy of the morphology of the hand of the “model”.

Modern human hand form and function have been thoroughly studied in different fields and with multiple aims, including work/ergonomics^6–11^, sports^12,13^, clinical^14,15^, forensic^16,17^ and personal identification^18–21^, biometry^21–23^, paleoanthropology, archaeology^24–31^ and other fields.

For biological sex^30,32,33^ and age ^27,30,32,34^ estimation from hand morphology, the most common approach has been conventional morphometrics, i.e. the study of form based on linear measurements, angles, ratios, etc^6,8,11,35–37^. Conventional morphometric approaches to archaeological hand stencils have consistently used different linear measurements and indexes (mostly ‘Manning Index’^38,39^, which is based on a sexually-based differentiation in the length of the 2nd and 4th digits due to fetal exposure to hormones) to estimate the biological sex^31,40,41^ of the individuals who left these images on the cave walls.

Despite the indisputable relevance of such studies, conventional morphometrics are affected by some limitations that led to the development of other morphometric approaches, such as Geometric Morphometrics (GM)^42,43^. GM is now a standard tool to examine morphology and its covariance with other underlying variables in multiple fields of research, such as biology, entomology, anatomy, biomechanics, palaeoanthropology, and bioanthropology^44–49^. In spite of its potential, few studies have actually used GM to infer the biological sex or/and age of living humans from their own hand morphology. The few studies that have employed this approach use a more or less standardized position^50–52^ and none of them has explored the actual archaeological record to examine the relevance of variations in relative finger positions on ensuing analyses.

**Figure 1:**
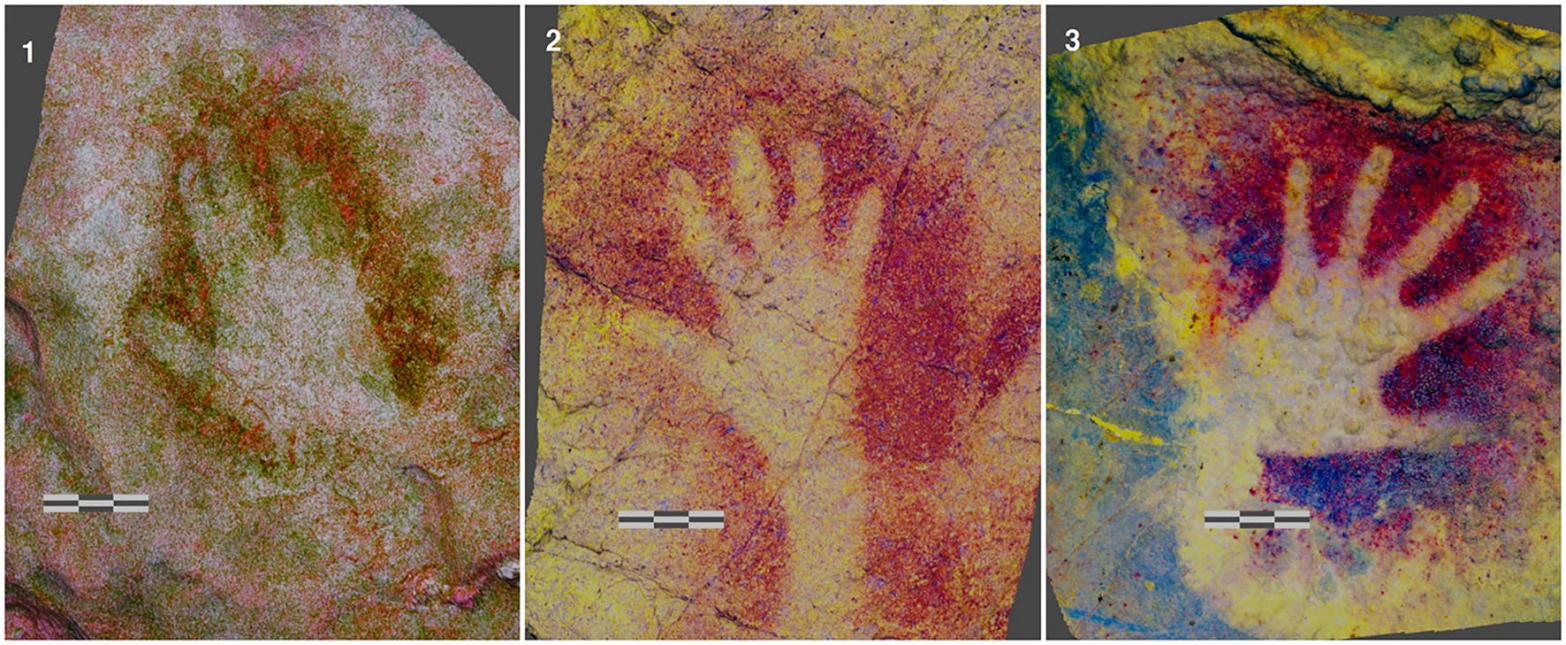
Colour alteration: D-stretch. 1.- Garma zone VII Hand 15; 2.- Fuente del Salín. Hand 6 Panel 2; 3.- Castillo Hand panel Hand 20. Models by: Handpas Project.

A meticulous analysis of the archaeological record reveals a considerable variability in hand positions on the wall, ranging from fully extended hands to nearly closed ones, with all intermediate configurations represented (see Fig. 1).Such variation in relative finger position may obscure an assessment of morphological differences between individuals because GM uses the spatial relationship between Landmark (LM) coordinates^44,53–55^. Thus, changing the position of fingers may induce changes in LM configurations that are larger than differences between individuals. Indeed, the potential problems that this archaeological variability could pose have not been formally addressed before^51^.

This present study investigates the statistical effect of the variability in relative hand position when conducting a GM study as well as identifying and describing the associated factors, with the ultimate objective of characterizing archaeological blown hand stencils. For that, we test the null hypothesis (H0), which states there are no significant morphological differences between different hand positions and those between subjects.

## Materials and methods

### Materials

2D left-hand scans were collected from 70 living adults of known biological sex and age (balanced sex sample of 35 biological females and 35 biological males; > 20 years of age, following Bogin & Smith, 1996^56^). The hands were digitized together with a scale using an HP Officejet Pro 8600 Plus contact scanner, which provides 300 dpi scans in jpeg format.

**Figure 2:**
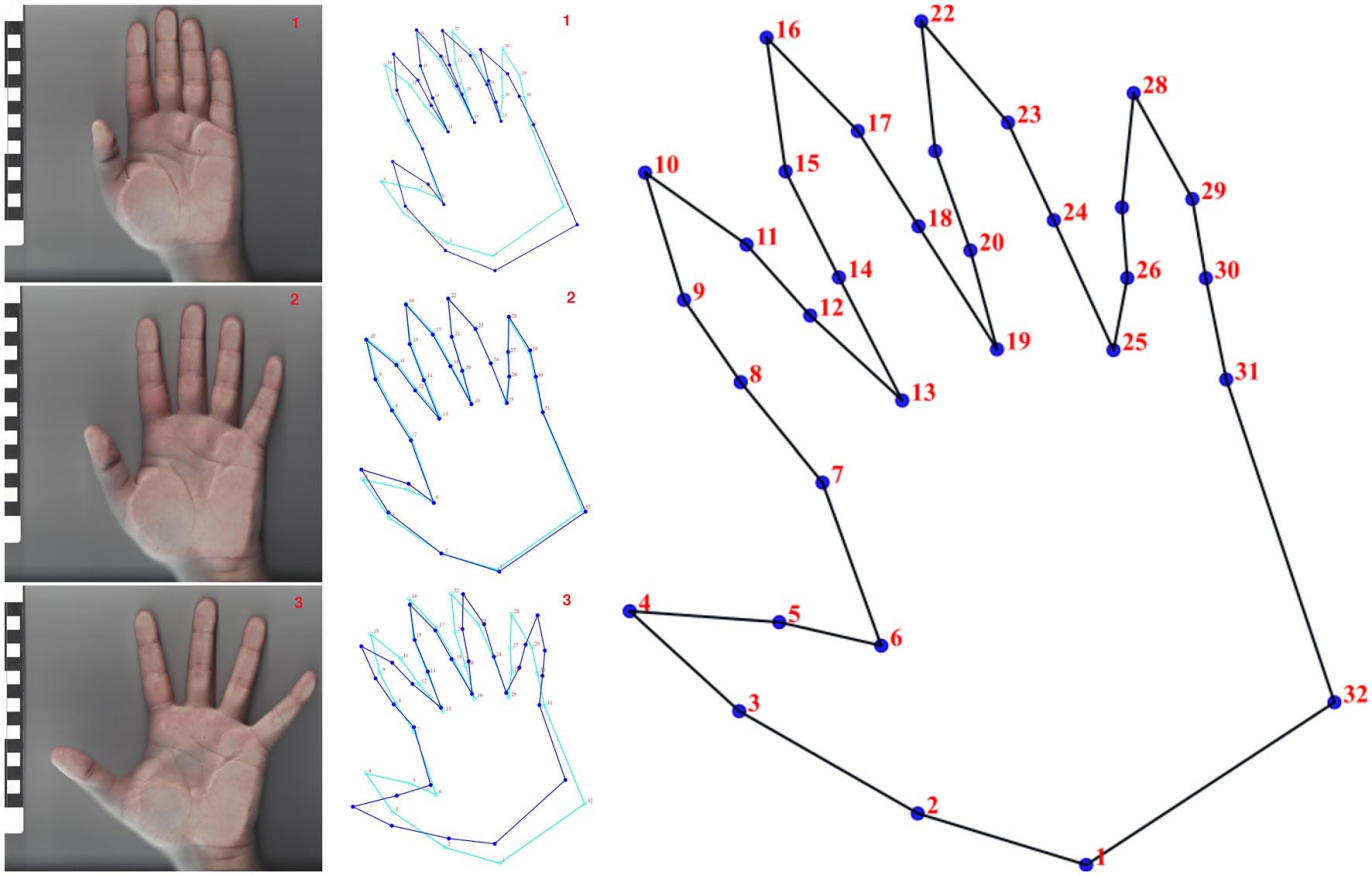
On the left: Standardized positions 1, 2, and 3 for an individual. In the middle: corresponding wireframes (light blue: mean shape; dark blue: individual shape) and landmark points. On the right: landmark template.

A scanning protocol established three different standardized hand positions (Fig. 2):

1. “Closed hand”, i.e., fingers fully extended but as close as possible (adducted) although not touching;
2. “Natural position”, i.e., fingers fully extended and semi-spread apart (abducted);
3. “Fully open”, fingers fully extended and abducted.

A scan of each position was obtained for all the participants, totalling three scans per individual. Thus, 210 (3 × 70) images were acquired.

None of the participants suffered from any pathologies affecting the limbs, amputations or deformities of the fingers and/or hands. Only left hands were scanned in the study, as this laterality is documented more in the archaeological record than right hands (L =  ~ 70%; R =  ~ 30%).

This experiment has been approved by the Ethics Committee of Cantabria University (Cod. CE Tesis 03/2021) following all the values and regulations for data protection and anonymity. In turn, we confirm that all steps of the experimental process have been carried out in accordance with the relevant guidelines and regulations. Finally, we confirm that informed consent was obtained from all subjects and/or their legal guardian(s) for the collection, processing and publication of the data, as well as that no other personal information has been collected but the strictly necessary for the study (biological sex and age).

### Methods

Thirty-two 2D conventional landmarks (LM) were acquired from the digital images of each digitization. Data were collected directly from the hand scans using TPSdig software. The LMs were located in the main anatomical reference points of the hand and enable a detailed size and shape morphological analysis of the hand (Fig. 2, Table 1). To that end, each scan was loaded into TPSdig2^®^^42^ and the corresponding LM coordinates were collected from each stencil image. The 2D landmark system can show sufficiently strong differentiation to test the null hypothesis (H0) and serves as a basis for a future study on parietal art which, given its nature, precludes a 360-degree view. All LM coordinates were then submitted to a Generalized Procrustes Analysis to remove the effects of translation, rotation, and scaling from the raw coordinates and to produce size and shape variables^55^.

**Table 1:** Corresponding landmark system formed by 32 conventional landmarks and their biological definition.

Landmark number	Landmark definition
1	Wrist and thumb junction point
2	Fold of the thumb joint on the medial side
3	Point of the distal palmar crease on the medial side
4	The central point of the fingertip (thumb f.)
5	Point of the distal palmar crease on the lateral side
6	The central point of the thumb joint corner
7	Digital-palmar crease on the medial side
8	The proximal digital crease of the index finger on the medial side
9	The distal digital crease of the index finger on the medial side
10	The central point of the fingertip (index f.)
11	The distal digital crease of the index finger on the lateral side
12	The proximal digital fold of the index finger on the lateral side
13	The central point of the 2nd finger joint corner
14	The proximal digital crease of the middle finger on the medial side
15	The distal digital crease of the middle finger on the medial side
16	The central point of the fingertip (middle f.)
17	The distal digital crease of the middle finger on the lateral side
18	The proximal digital crease of the middle finger on the medial side
19	The central point of the 3rd finger joint corner
20	The proximal digital crease of the ring finger on the medial side
21	The distal digital crease of the ring finger on the medial side
22	The central point of the fingertip (ring f.)
23	The distal digital crease of the ring finger on the lateral side
24	The proximal digital crease of the ring finger on the lateral side
25	The central point of the 4th finger joint corner
26	The proximal digital crease of the little finger on the medial side
27	The digital crease of the little finger on the medial side
28	The central point of the fingertip (little f.)
29	The digital crease of the little finger on the lateral side
30	The proximal digital crease of the little finger on the lateral side
31	Digital-palmar crease on the lateral side
32	Point of attachment between the little finger and the wrist

First, Shapiro–Wilk test was run to test the normality of the dataset. Resulting value concludes no normality data (p < 0.05), so subsequent tests have therefore been adapted to the nature of the data. Subsequently, Centroid Size (CS), defined as the square root of the sum of squared distances of all landmarks from their centroid^55^, was employed to assess the impact of hand position on object size. Subsequently, centroid size was measured and categorized based on hand position (as described above). To further explore the intricacies of size and its correlation with shape, an allometric study^57,58^ was conducted. A multivariate regression analysis was employed with shape (represented by Procrustean coordinates) as the dependent variable and size (Centroid Size) as the independent one with 100 permutations.

Afterwards, Procrustes distances between individuals were computed for each position, representing inter-individual distances. These were then compared to Procrustes distances of the same individuals but with different hand positions, representing intra-individual differences. The results were organized based on hand position and subsequently visualized using boxplots. If the observed differences between contrasting intra-individual hand positions are larger than those between individuals, it suggests that the former has a more substantial impact on the morphological analysis. This, in turn, hinders the comparison of hand stencils in different positions.

Additionally, we conducted two distinct Principal Component Analyses (PCAs) to scrutinize both the data and explanatory variables of shape, stratifying the samples by their biological sex. The initial PCA was based on the variance–covariance matrix after a Procrustes superimposition. Our examination focused on the first two Principal Components, guided by the scree plot to identify the components with the most substantial variance explanation (Supp. Fig. 1).

In turn, a second Principal Component Analysis was performed on the residual data from first PCA derived from a dummy regression between Procrustes coordinates and position of the covariate (covariate: Position 1 = -1; Position 2 = 0; Position 3 =  + 1). This procedure aims to mitigate the influence of relative finger position as this factor has been demonstrated to contribute a significant percentage of variability, potentially obscuring other influential factors.

Due to the non-normal distribution of the data and the repetitive nature of cases, the Friedman test was employed to examine potential differences in both size (evaluated through centroid size) and shape (evaluated through PC scores) among groups, defined by finger position. This statistical analysis was conducted using Past^59^, with subsequent pairwise Wilcoxon post-hoc tests for detailed comparisons between groups.

Finally, it’s crucial to acknowledge that various variables such as age, biological sex, or musculature may potentially influence hand shape, and their impact might be obscured by the high percentage explained by hand movement. In-depth studies on biological sex and ontogeny variables have been extensively explored where each element of the hand was individually examined^60^. These studies revealed distinct results in biological sex differences observed from childhood onwards (+ 7 years) in terms of size and from youth onwards in terms of shape (+ 10/12 years). Consequently, we have chosen to briefly explore the variable of biological sex in this work to illustrate the trends that may be obscured by the dominant influence of hand movement. To test whether the difference between biological males and females is statistically significant, the Friedman two-sample test was run in all mentioned statistical procedures, both Centroid Size data and PCA Scores.

The intra-observer error study was performed by taking five measurements of the same scan^61^. The intra-observer error was accepted after testing that the largest Procrustes distance within the measurements of the same individual (0.013819) is smaller than the smallest Procrustes distance between different individuals (0.020328)^61^.

GM data collection and analyses in this study were performed in tpsDig, tpsRelw, and MorphoJ, which are freely available at the SUNY Stony Brook Morphometrics website (http://life.bio.sunysb.edu/morph/). Statistical testing was undertaken in MorphoJ and Past^59^.

## Results

**Figure 3:**
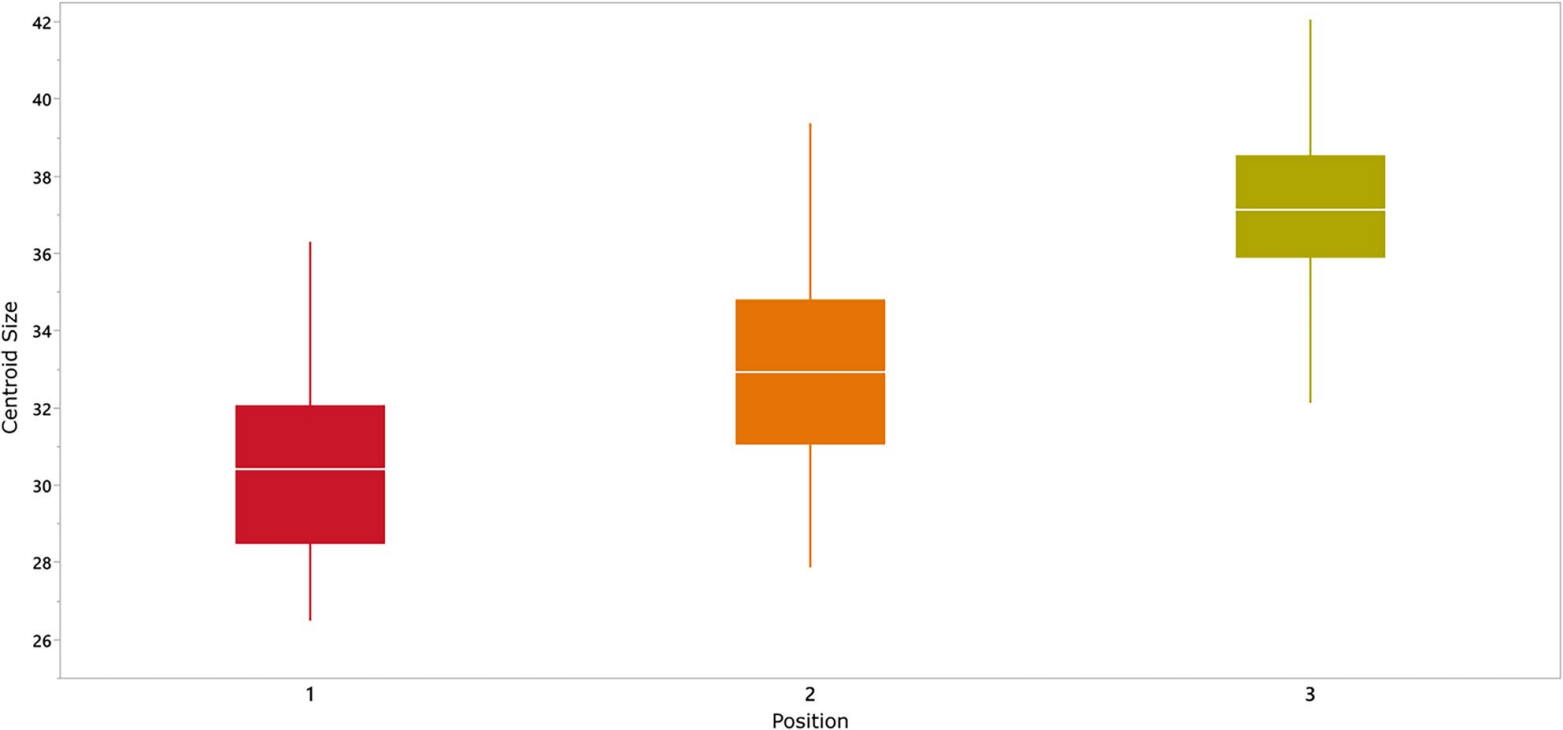
Boxplot with the Centroid Size of each group. From left to right: 1, 2, 3. Detailed CS values in Supp. Table 1.

The results reveal a clear size distinction associated with hand position. Specifically, Centroid Size (CS) shows an incremental pattern from Position 1 to Position 3 (mean of Pos. 1 = 30.52, Pos. 2 = 32.98, and Pos. 3 = 36.93), correlating with finger abduction (refer to Fig. 3). The Friedman test indicates a statistically significant difference among their medians (*p* < 3.694E − 28). Additionally, post-hoc Wilcoxon pairwise tests demonstrate a significant size difference between all groups (P1-P2 p = 5.3188E − 12; P2-P3 p = 5.1041E − 12; P1-P3 p = 3.5595E − 13).

**Figure 4:**
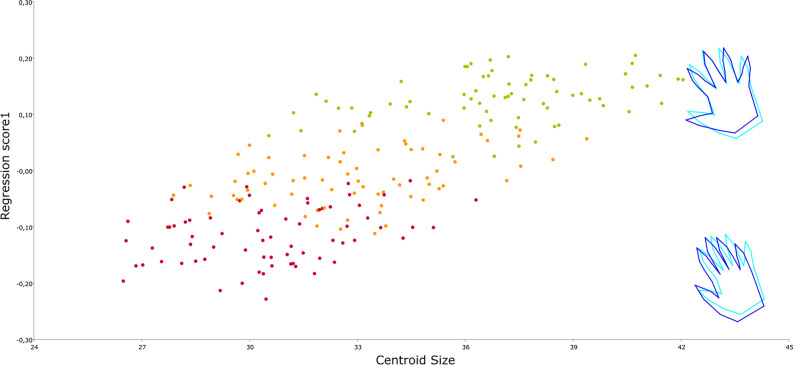
Allometry regression being shape the dependent variable and centroid size the independent one. Red = Position 1; Orange = Position 2; Green = Position 3. Wireframes regression factor showed in ± 5.

Regarding allometry, results indicate a predicted percentage of 40.46%, which implies that a significant portion of the shape is explained by size (Fig. 4). The regression scores exhibit a significant difference among the three groups according to the Friedman test and Wilcoxon pairwise comparisons. As supported by Fig. 4, individuals' shapes are strongly correlated with their size changes. The analysis reveals that hands with a smaller Centroid Size correspond to closed hands, while larger Centroid Sizes correspond to open hands. Individuals are therefore organized based on their degree of openness: 1, 2, or 3.

**Figure 5:**
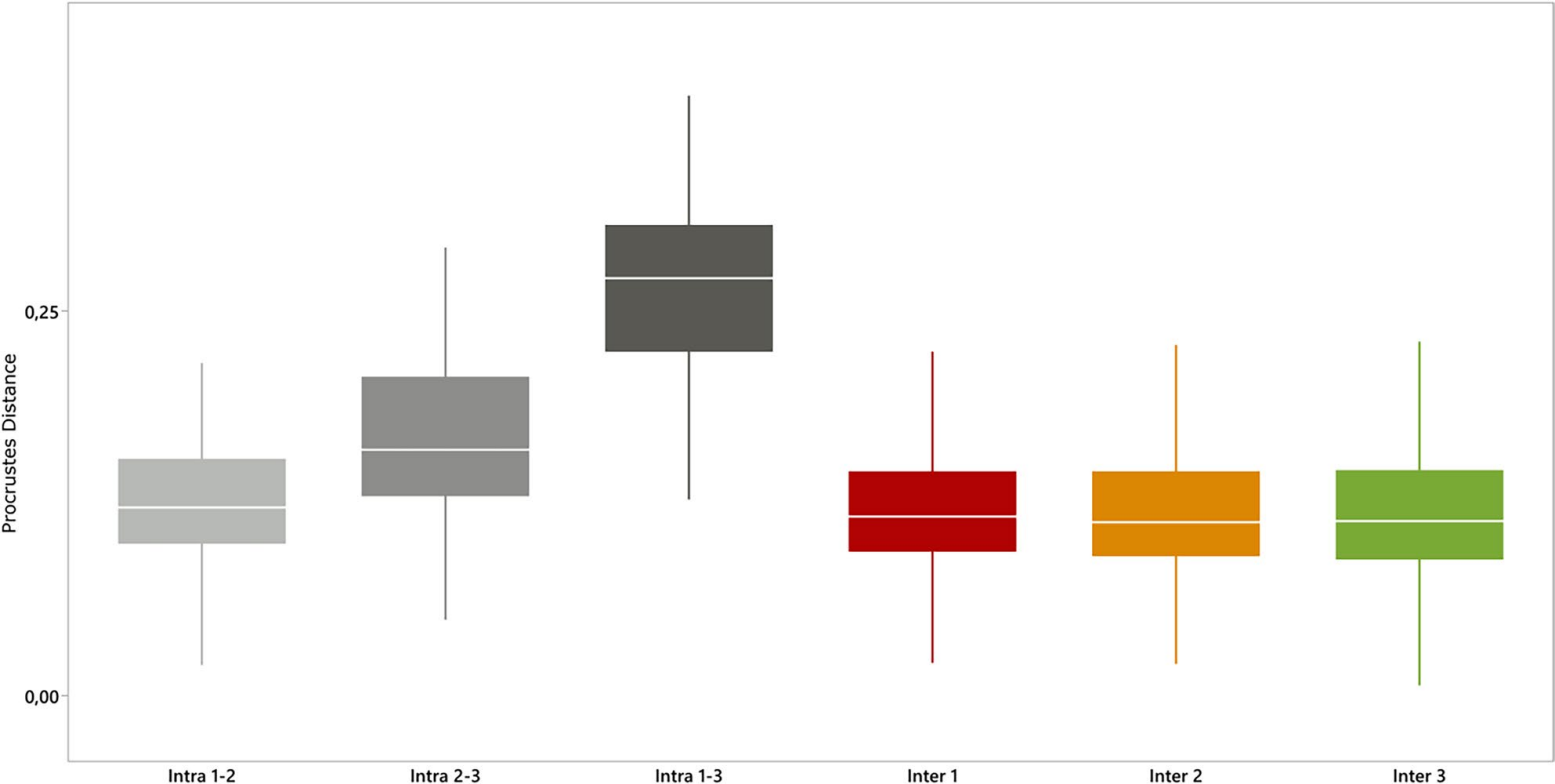
Boxplot of the values of inter-individual and intra-individual Procrustes distances. From left to right: intra-individual distances between 1–2; intra-individual distances 2–3; intra-individual distances 1–3; inter-individual in 1; inter-individual in 2; inter-individual in 3.

Figure 5 shows that inter-individual Procrustes distances are consistent across hand positions and lower than intra-individual differences. The latter are lowest between hand positions 1 and 2 (i.e., between adducted and semi-abducted fingers) and largest between positions 1 and 3 (i.e., between adducted and fully abducted fingers). This implies that the statistical difference within the same hand (intra-individual average Procrustes distance: Position 1–2 = 0.137; Position 2–3 = 0.191; Position 1–3 = 0.292) is greater than the difference existing between hands of different individuals (inter-subject average Procrustes distance: 1 = 0.122; 2 = 0.142; 3 = 0.165).

**Figure 6:**
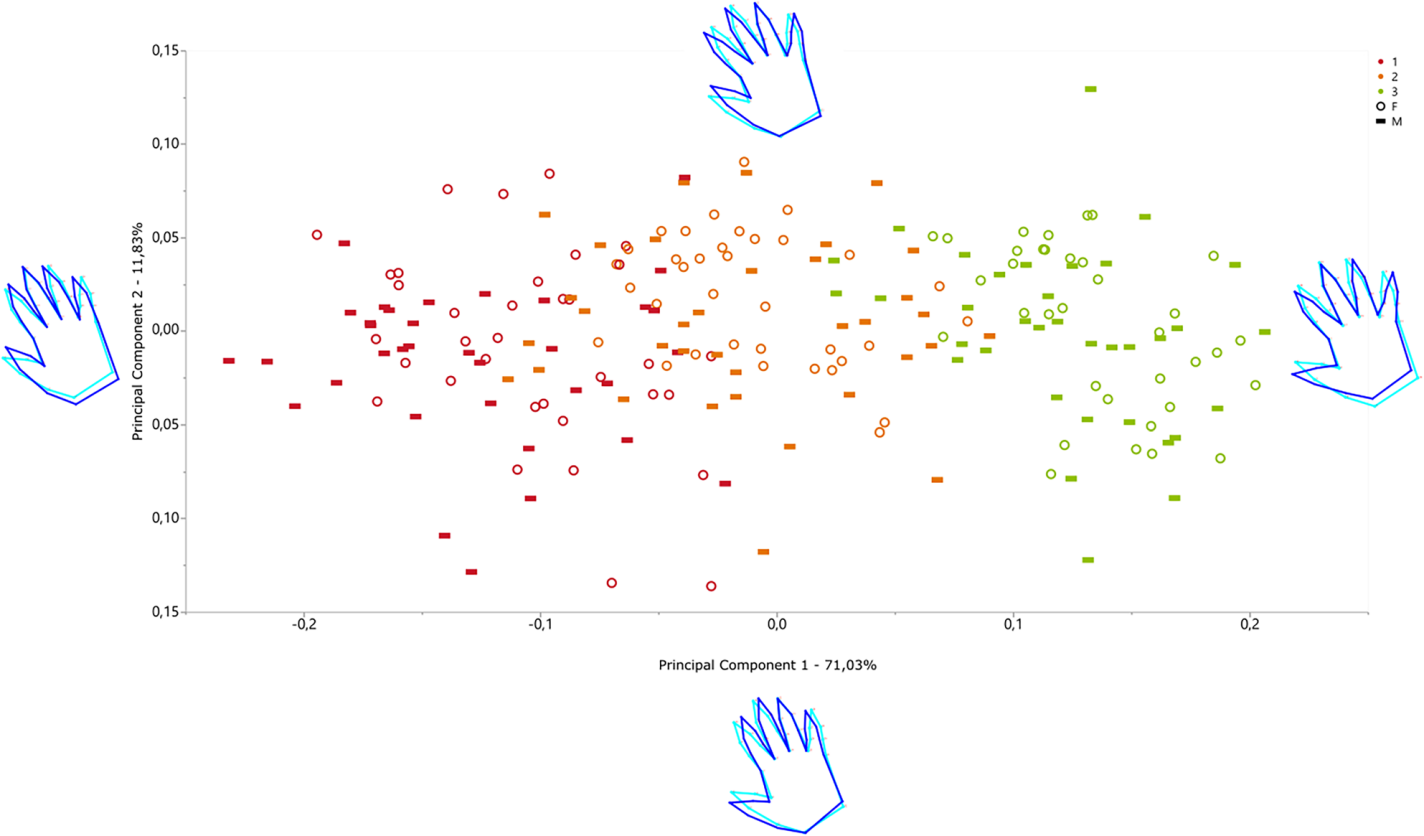
Shape PCA of the complete record (1 in red, 2 in orange and 3 in green) with the corresponding wireframe on PC1 ± 1 and PC2 ± 1 scores. Detailed PC Scores and analysis in Supp. Tables 3–5. Females: circle; Males: hyphen.

Additionally, we perform a PCA on the covariance matrix after a Procrustes superimposition. In this first PCA, we observe the individual arrangement of groups from left to right, associated with decreasing to increasing finger openness (i.e., abduction), as shown by the wireframes (Fig. 6). Notably, 82.86% of the variance is elucidated by the first two principal components. The Friedman test indicates a significant difference among the medians of the three positions in both PC1 and PC2 (p = 7.1694E-30 and 0.0015). Furthermore, Wilcoxon pairwise tests disclose significant differences between all groups in PC1 (p < 0.01). However, in PC2, no significant differences are discerned between Position 2 and Position 3 (p = 0.1056).

**Figure 7:**
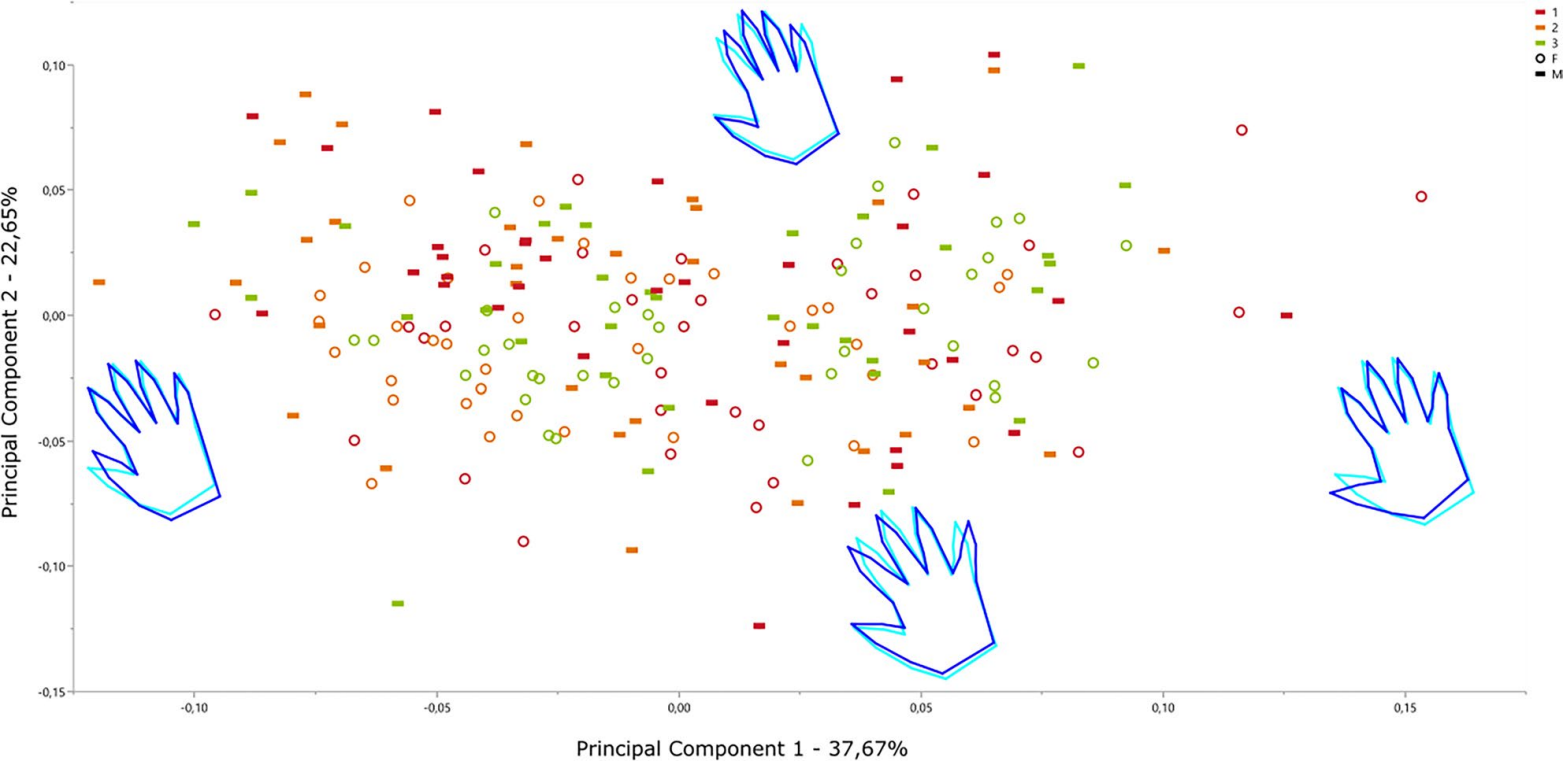
Shape PCA of the complete record (1 in red, 2 in orange and 3 in green) excluding position/movement. Detailed PC Scores and values in Supp. Females: circle; Males: hyphen.

Then trough a second Principal Component Analysis (Fig. 7), run on resulted data from a dummy regression on the residuals data from the first principal component analysis, we determine that 58.13% of the variance in shape can be explained by movement. The Friedman test indicates significant differences among the medians of the three groups in PC1 (p = 1.5255E − 05). Finally, according to Wilcoxon pairwise tests, positions 1 and 3 are not distinguishable in PC1 (p = 0,692), and none of the three positions are distinguishable from each other PC2 (p = 0.88568). As it can be slightly noticed in the wireframes even after removing the effect of the finger extension category (1, 2, 3), PC1 and PC2 are not completely free from its influence.

Finally, in relation to biological sex, through Friedman two-sample test, statistical differentiation in size (Centroid Size) has been identified, both in the analysis of the overall sample and in each position. On the contrary, regarding the aspect of shape no statistical differences were observed between males and females in either of the two Principal Component Analyses (PC Scores in PC1 and PC2), whether examining all positions collectively or individually within each position.

## Conclusion and discussion

This article explores the application of Geometric Morphometrics (GM) in the analysis of prehistoric hand stencils, highlighting its potential in a relatively unexplored field within rock art studies. Palaeolithic hand stencils corpus reveals significant variability in hand positions in rock art, ranging from fully extended hands to partially closed ones. This variability poses challenges for morphometrics and underscores the need to formally address it. Through this work it is demonstrated that relative finger positions variable affects significantly the results and should be specifically addressed. So that, the null hypothesis (H0) proposed at the beginning of the study is rejected as the results show that the difference between hands of the same individual in its three positions is, in fact, greater than that between the hands of different individuals in each of the positions.

On one hand, results reveal a significant size distinction associated with hand position, indicating a predictable relationship between finger abduction and centroid size. The regression of Procrustes Coordinates against Centroid Size reinforces the substantial percentage of shape explained by size, with hands of smaller centroid sizes corresponding to closed positions and larger ones to open positions. Thus, depending on the questions, centroid size may not represent the best option when studying the parameter of size under conditions comparable to those of this study. On the other, Procrustes distances and PCA analyses highlight the substantial influence of relative finger positions on morphology. PCA results emphasized the influence of hand movement on shape variation, concluding 58.13% of the shape variation could be explained by relative finger position.

Regarding biological sex, it has been documented a statistical differentiation in size (Centroid Size), both in the analysis of the overall sample and in each position, where on shape no statistical differences were observed between males and females in either of the two Principal Component Analyses (PC Scores in PC1 and PC2). These results contradict those obtained in Fernández-Navarro et al. 2024^60^ where the difference in size between biological males and females is statistically discernible from ages 3–7 and in shape from ages 7–13.

On the other hand, the efficacy of the palm has been previously pointed out and discussed, arguing that it is the most appropriate element for the evaluation of sex through human hands^62,50^, especially since it is not as exposed to changes in hand posture as are the fingers^52^. This parameter, along with others related to sexual dimorphism of the hands, will be analysed in a dedicated article^60^.

We can conclude that the combined analysis of hand elements is masking other potential variables, such as sexual dimorphism. So, results in this paper do not imply a real absence of differentiation between the biological sexes. Instead, it suggests that, given the inclusive nature of the hand elements in the current sample, any such distinctions are not visual and statistical apparent.

This highlights the inaccuracies in classifying and characterizing an individual through hand scanning if the variable of movement/positioning is not homogenized beforehand or, alternatively, extracted through the independent analysis of its elements^51,52^. Thus, the establishment of a standardized system in data collection or the analysis of independent elements should be considered when examining previous studies in a wide variety of disciplines in which the object of study is composed of moveable structures^60^. In short, this article is a first step towards the correct use of GM in contexts related to archaeological materials, especially those containing postural information which, as we have shown, has a decisive influence on any biometric approach.

Our results showing the meaningful impact of hand/finger position are thus tracking first, are foremost, movement, and so are consistent with previous research using GM to examine motion of, e.g., fish feeding systems^63,64^, human gape cycle^65^, heart cycle^66^, posture and limbs^67^ or breathing^68,69^. In such studies, sequential postural changes are used to assess motion trajectories in multivariate shape space^68^.

Finally, we would like to highlight the experimental nature of this work. This data cannot be directly extrapolated to archaeological contexts and conditions; instead, it serves as an experimental study guiding the design of data collection and the corresponding analysis framework. This clarification will help ensure a comprehensive understanding of the study's scope and its applicability to real hand findings within the context of Paleolithic art.

### Supplementary Information

Supplementary Information.

## Data Availability

Corresponding raw-measurement dataset is not publicly available due to personal privacy reasons but are available from the corresponding author on reasonable request. Additionally, a comprehensive supplementary information file collecting summary statistical description/information generated by the sample analysis is included.
