# Supplementary material for: Exploring the utility of Geometric Morphometrics to analyse prehistoric hand stencils
Source: Sci Rep. 2024 Mar 15;14:6336. doi: 10.1038/s41598-024-56889-3 (PMC10943054; doi:10.1038/s41598-024-56889-3)
Supplement: Supplementary file 1 — Supplementary Information. [file 41598_2024_56889_MOESM1_ESM.docx]

**Title: Exploring the utility of Geometric Morphometrics to analyse archaeological/Upper Palaeolithic hand stencils ( DECIDIR SI UPPER P O ARCHAEOLOGICAL).**

**Authors:**

V. Fernández Navarro. Instituto Internacional de Investigaciones Prehistóricas de Cantabria (IIIPC), Universidad de Cantabria, Gobierno de Cantabria, Santander, Avenida de los Castros s/n, 39005, Santander, Spain* [veronica.fernandezn@unican.es](mailto:veronica.fernandezn@unican.es)

R. M. Godinho. Interdisciplinary Center for Archaeology and Evolution of Human Behaviour (ICArHEB), University of Algarve, Faculdade das Ciências Humanas e Sociais, Faro, Portugal. [rmgodinho@ualg.pt](mailto:rmgodinho@ualg.pt)

D. García Martínez. Physical Anthropology Unit, Department of Biodiversity, Ecology, and Evolution, Faculty of Biological Sciences, Complutense University of Madrid, Madrid, Spain. [dangar29@ucm.es](mailto:dangar29@ucm.es)

D. Garate Maidagan**.** Instituto Internacional de Investigaciones Prehistóricas de Cantabria (IIIPC), Universidad de Cantabria, Gobierno de Cantabria, Santander, Avenida de los Castros s/n, 39005, Santander, Spain [diego.garate@unican.es](mailto:diego.garate@unican.es)

**Funding information:**

The author V. Fernandez-Navarro ´ is beneficiary of a “Concepcion ´ Arenal” 2020–21 pre-doctoral contract at the University of Cantabria for research toward the doctoral thesis titled “From the hand to the mind: demography and semiotics of Palaeolithic artists through the representation of hands”.

RM Godinho is funded by Fundação para a Ciência e a Tecnologia (FCT; contract reference 2020.00499.CEECIND and R&D project reference 2022.07737.PTDC)

The present study has been conducted as part of the research project “Before Art: social investment in symbolic expressions during the Upper Palaeolithic (B-Art)” (PID 2019-107262 GB-I00), funded by the Ministry of Science, Innovation, and Universities (MCIN/ AEI /10.13039/ 501100011033), PI: Diego Garate.

**Number of figure**s: 6

**Number of tables:** 1

**SUPPLEMENTARY INFORMATION:**

**Fig. 1. Principal Component Analysis resulting Scree Plot.**


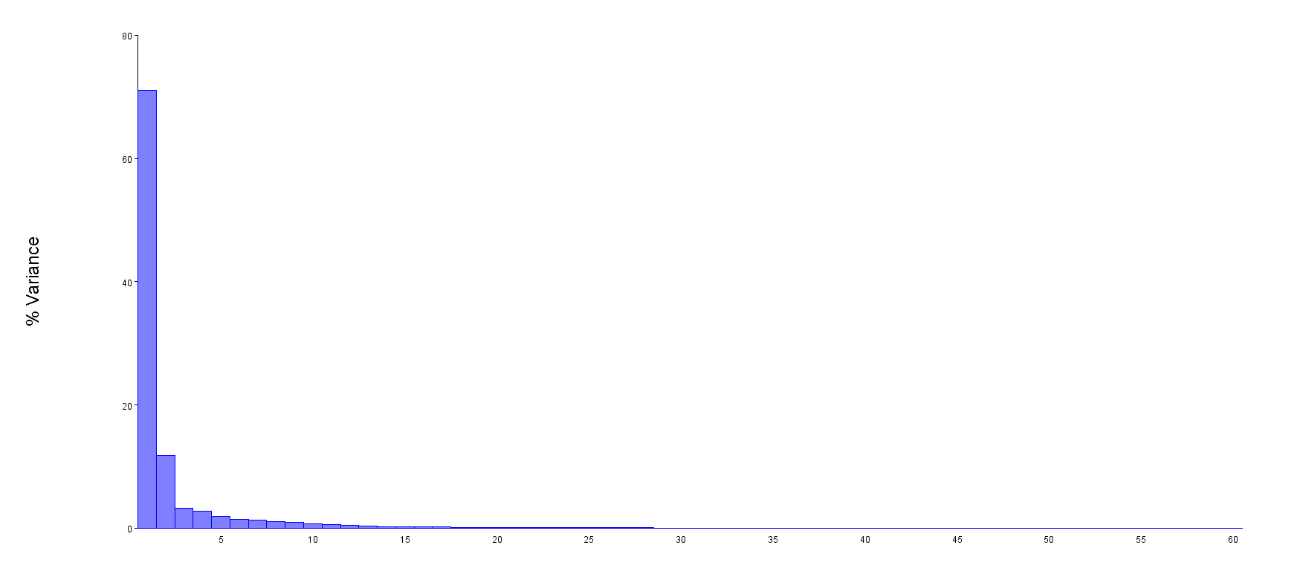


**Tab. 1. Centroid Size values:**

| Id | Sex | Position | Centroid Size |
| --- | --- | --- | --- |
| Ind6.F.1 | F | 1 | 28,2132264 |
| Ind5.F.1 | F | 1 | 27,72600303 |
| Ind14.F.1 | F | 1 | 27,77296242 |
| Ind7.M.1 | M | 1 | 35,11068061 |
| Ind9.F.1 | F | 1 | 32,24415566 |
| Ind10.F.1 | F | 1 | 32,74357989 |
| Ind11.M.1 | M | 1 | 30,3946433 |
| Ind12.M.1 | M | 1 | 34,46738395 |
| Ind1.F.1 | F | 1 | 27,2923129 |
| Ind2.F.1 | F | 1 | 29,88435842 |
| Ind3.F.1 | F | 1 | 28,49851384 |
| Ind4.F.1 | F | 1 | 27,5498414 |
| Ind13.F.1 | F | 1 | 26,61514239 |
| Ind20.M.1 | M | 1 | 30,36596061 |
| Ind15.M.1 | M | 1 | 32,78019637 |
| Ind16.F.1 | F | 1 | 31,00679607 |
| Ind19.F.1 | F | 1 | 29,99465817 |
| Ind18.M.1 | M | 1 | 31,3902253 |
| Ind22.F.1 | F | 1 | 29,72316033 |
| Ind25.F.1 | F | 1 | 30,58581285 |
| Ind26.F.1 | F | 1 | 33,64480117 |
| Ind27.F.1 | F | 1 | 30,26334281 |
| Ind29.F.1 | F | 1 | 28,33414 |
| Ind8.M.1 | M | 1 | 31,60711411 |
| Ind24.M.1 | M | 1 | 31,48801909 |
| Ind30.F.1 | F | 1 | 32,02126075 |
| Ind31.M.1 | M | 1 | 32,59132662 |
| Ind32.M.1 | M | 1 | 31,04437482 |
| Ind33.M.1 | M | 1 | 31,61634903 |
| Ind34.F.1 | F | 1 | 27,90138521 |
| Ind35.F.1 | F | 1 | 26,56388818 |
| Ind36.M.1 | M | 1 | 33,05261512 |
| Ind37.F.1 | F | 1 | 28,17981474 |
| Ind38.M.1 | M | 1 | 32,91879532 |
| Ind39.M.1 | M | 1 | 31,20934957 |
| Ind40.F.1 | F | 1 | 31,9390186 |
| Ind41.F.1 | F | 1 | 28,11003189 |
| Ind42.M.1 | M | 1 | 31,14384171 |
| Ind44.F.1 | F | 1 | 30,22792792 |
| Ind45.F.1 | F | 1 | 26,4867034 |
| Ind46.M.1 | M | 1 | 31,15554781 |
| Ind47.F.1 | F | 1 | 28,75358582 |
| Ind48.F.1 | F | 1 | 28,90793122 |
| Ind49.F.1 | F | 1 | 28,40261402 |
| Ind50.M.1 | M | 1 | 34,26674454 |
| Ind57.F.1 | F | 1 | 27,83383657 |
| Ind59.M.1 | M | 1 | 30,32784325 |
| Ind58.F.1 | F | 1 | 29,92031125 |
| Ind53.M.1 | M | 1 | 32,35864027 |
| Ind52.F.1 | F | 1 | 29,23284551 |
| Ind51.M.1 | M | 1 | 30,62011548 |
| Ind54.F.1 | F | 1 | 27,02784966 |
| Ind55.M.1 | M | 1 | 31,94839105 |
| Ind56.F.1 | F | 1 | 28,99886602 |
| Ind60.M.1 | M | 1 | 31,28162714 |
| Ind62.F.1 | F | 1 | 28,35480436 |
| Ind61.F.1 | F | 1 | 26,83814366 |
| Ind64.M.1 | M | 1 | 30,38205634 |
| Ind63.M.1 | M | 1 | 34,53458181 |
| Ind65.M.1 | M | 1 | 32,31621301 |
| Ind67.M.1 | M | 1 | 29,18231926 |
| Ind68.M.1 | M | 1 | 30,455609 |
| Ind69.M.1 | M | 1 | 30,59836083 |
| Ind70.M.1 | M | 1 | 31,80177046 |
| Ind71.M.1 | M | 1 | 33,28277421 |
| Ind72.M.1 | M | 1 | 33,74080444 |
| Ind73.M.1 | M | 1 | 32,7088268 |
| Ind74.M.1 | M | 1 | 30,26528558 |
| Ind75.M.1 | M | 1 | 36,29490822 |
| Ind66.M.1 | M | 1 | 29,79350958 |
| Ind6.F.2 | F | 2 | 30,43390127 |
| Ind5.F.2 | F | 2 | 28,93587392 |
| Ind14.F.2 | F | 2 | 30,11738962 |
| Ind8.M.2 | M | 2 | 35,38729312 |
| Ind9.F.2 | F | 2 | 33,0213342 |
| Ind10.F.2 | F | 2 | 34,34121072 |
| Ind11.M.2 | M | 2 | 32,91145765 |
| Ind12.M.2 | M | 2 | 37,51965354 |
| Ind1.F.2 | F | 2 | 29,59634315 |
| Ind2.F.2 | F | 2 | 32,28300822 |
| Ind3.F.2 | F | 2 | 31,17117674 |
| Ind4.F.2 | F | 2 | 29,65270752 |
| Ind13.F.2 | F | 2 | 28,34704139 |
| Ind20.M.2 | M | 2 | 32,0218085 |
| Ind15.M.2 | M | 2 | 34,81163903 |
| Ind16.F.2 | F | 2 | 32,97624425 |
| Ind19.F.2 | F | 2 | 32,50815039 |
| Ind18.M.2 | M | 2 | 34,49080316 |
| Ind22.F.2 | F | 2 | 33,13484869 |
| Ind25.F.2 | F | 2 | 33,15669729 |
| Ind26.F.2 | F | 2 | 35,38523944 |
| Ind27.F.2 | F | 2 | 33,5719703 |
| Ind29.F.2 | F | 2 | 29,95363911 |
| Ind7.M.2 | M | 2 | 39,37222825 |
| Ind24.M.2 | M | 2 | 32,52900477 |
| Ind30.F.2 | F | 2 | 31,88518974 |
| Ind31.M.2 | M | 2 | 34,48234621 |
| Ind32.M.2 | M | 2 | 33,7215282 |
| Ind33.M.2 | M | 2 | 34,30811313 |
| Ind34.F.2 | F | 2 | 30,53388908 |
| Ind35.F.2 | F | 2 | 27,88289152 |
| Ind36.M.2 | M | 2 | 36,43355516 |
| Ind37.F.2 | F | 2 | 29,99589858 |
| Ind38.M.2 | M | 2 | 35,26815219 |
| Ind39.M.2 | M | 2 | 35,71083888 |
| Ind40.F.2 | F | 2 | 32,07648582 |
| Ind41.F.2 | F | 2 | 31,20943265 |
| Ind42.M.2 | M | 2 | 33,57182813 |
| Ind44.F.2 | F | 2 | 32,72713957 |
| Ind45.F.2 | F | 2 | 29,55188464 |
| Ind46.M.2 | M | 2 | 35,29096453 |
| Ind47.F.2 | F | 2 | 31,52867917 |
| Ind48.F.2 | F | 2 | 31,52632518 |
| Ind49.F.2 | F | 2 | 29,78150677 |
| Ind50.M.2 | M | 2 | 37,14908133 |
| Ind59.M.2 | M | 2 | 32,61949105 |
| Ind58.F.2 | F | 2 | 30,64015669 |
| Ind57.F.2 | F | 2 | 29,67914298 |
| Ind53.M.2 | M | 2 | 33,48052272 |
| Ind52.F.2 | F | 2 | 32,51568865 |
| Ind51.M.2 | M | 2 | 33,66465129 |
| Ind54.F.2 | F | 2 | 28,87480423 |
| Ind55.M.2 | M | 2 | 33,62316707 |
| Ind56.F.2 | F | 2 | 30,69617841 |
| Ind60.M.2 | M | 2 | 34,9951214 |
| Ind61.F.2 | F | 2 | 29,94221879 |
| Ind64.M.2 | M | 2 | 34,17021441 |
| Ind62.F.2 | F | 2 | 32,19112644 |
| Ind63.M.2 | M | 2 | 37,537667 |
| Ind65.M.2 | M | 2 | 35,20625246 |
| Ind67.M.2 | M | 2 | 31,87228491 |
| INd68.M.2 | M | 2 | 34,82646145 |
| Ind69.M.2 | M | 2 | 31,54681442 |
| Ind66.M.2 | M | 2 | 34,00414821 |
| Ind70.M.2 | M | 2 | 33,78490726 |
| Ind71.M.2 | M | 2 | 34,93768489 |
| Ind72.M.2 | M | 2 | 36,61270252 |
| Ind73.M.2 | M | 2 | 37,48154147 |
| Ind74.M.2 | M | 2 | 32,7239775 |
| Ind75.M.2 | M | 2 | 38,43510907 |
| Ind14.F.3 | F | 3 | 32,47099847 |
| Ind6.F.3 | F | 3 | 33,12368453 |
| Ind5.F.3 | F | 3 | 31,42839083 |
| Ind7.M.3 | M | 3 | 41,4573423 |
| Ind8.M.3 | M | 3 | 36,79513711 |
| Ind9.F.3 | F | 3 | 35,96369189 |
| Ind10.F.3 | F | 3 | 37,81946261 |
| Ind11.M.3 | M | 3 | 36,58607975 |
| Ind12.M.3 | M | 3 | 39,7350286 |
| Ind4.F.3 | F | 3 | 34,46002818 |
| Ind1.F.3 | F | 3 | 32,84016994 |
| Ind2.F.3 | F | 3 | 38,28215463 |
| Ind3.F.3 | F | 3 | 35,96211332 |
| Ind13.F.3 | F | 3 | 31,21960214 |
| Ind20.M.3 | M | 3 | 37,6823804 |
| Ind15.M.3 | M | 3 | 37,62547833 |
| Ind16.F.3 | F | 3 | 34,98219141 |
| Ind19.F.3 | F | 3 | 35,9880289 |
| Ind18.M.3 | M | 3 | 37,27912291 |
| Ind22.F.3 | F | 3 | 37,19630358 |
| Ind25.F.3 | F | 3 | 38,45499533 |
| Ind26.F.3 | F | 3 | 41,91780387 |
| Ind.27.F3 | F | 3 | 37,21246605 |
| Ind29.F.3 | F | 3 | 33,90838652 |
| Ind24.M.3 | M | 3 | 35,65124901 |
| Ind30.F.3 | F | 3 | 37,10622253 |
| Ind31.M.3 | M | 3 | 41,41935892 |
| Ind32.M.3 | M | 3 | 38,16863379 |
| Ind33.M.3 | M | 3 | 37,84417204 |
| Ind34.F.3 | F | 3 | 34,21296946 |
| Ind35.F.3 | F | 3 | 30,5318082 |
| Ind36.M.3 | M | 3 | 38,54002189 |
| Ind37.F.3 | F | 3 | 32,12621808 |
| Ind38.M.3 | M | 3 | 38,5977929 |
| Ind39.M.3 | M | 3 | 38,27915245 |
| Ind40.F.3 | F | 3 | 37,18814981 |
| Ind42.M.3 | M | 3 | 37,4740825 |
| Ind41.F.3 | F | 3 | 36,15833817 |
| Ind44.F.3 | F | 3 | 36,39715172 |
| Ind45.F.3 | F | 3 | 33,35999974 |
| Ind46.M.3 | M | 3 | 40,45186406 |
| Ind47.F.3 | F | 3 | 34,36157619 |
| Ind48.F.3 | F | 3 | 36,04901617 |
| Ind49.F.3 | F | 3 | 36,68993483 |
| Ind50.M.3 | M | 3 | 42,05016491 |
| Ind58.F.3 | F | 3 | 32,91539804 |
| Ind59.M.3 | M | 3 | 36,49690733 |
| Ind57.F.3 | F | 3 | 31,85013636 |
| Ind53.M.3 | M | 3 | 37,48868454 |
| Ind52.F.3 | F | 3 | 36,64266967 |
| Ind51.M.3 | M | 3 | 37,41160306 |
| Ind54.F.3 | F | 3 | 33,33026007 |
| Ind55.M.3 | M | 3 | 36,8043424 |
| Ind56.F.3 | F | 3 | 36,29075827 |
| Ind60.M.3 | M | 3 | 39,46181683 |
| Ind63.M.3 | M | 3 | 39,83343587 |
| Ind61.F.3 | F | 3 | 36,15531691 |
| Ind64.M.3 | M | 3 | 38,9869392 |
| Ind62.F.3 | F | 3 | 36,73697121 |
| Ind65.M.3 | M | 3 | 39,22454438 |
| Ind67.M.3 | M | 3 | 36,39802712 |
| Ind68.M.3 | M | 3 | 38,4777208 |
| Ind69.M.3 | M | 3 | 39,33966827 |
| Ind66.M.3 | M | 3 | 36,71201572 |
| Ind70.M.3 | M | 3 | 37,94803248 |
| Ind71.M.3 | M | 3 | 40,64244185 |
| Ind72.M.3 | M | 3 | 40,72960907 |
| Ind73.M.3 | M | 3 | 41,05269185 |
| Ind74.M.3 | M | 3 | 40,63606339 |
| Ind75.M.3 | M | 3 | 40,55231078 |

Table 1. Centroid Size Value of each individual of the sample indicating sex and position.

**Tab. 2. Centroid Size analysis:**

Kruskal-Wallis between 3 positions: p (same): 2,776E-24*

Dun´s post hoc:

|  | P-1 | P-2 | P-3 |
| --- | --- | --- | --- |
| P-1 |  | 1,884E-05* | 3,649E-25* |
| P-2 | 1,884E-05* |  | 1,166E-09* |
| P-3 | 3,649E-25* | 1,166E-09* |  |

Supp. Table 2. Centroid Size analysis

**Tab. 3. General PCA Eigenvalues** (Fig. 5)**:**

| Eigenvalues | % Variance | Cumulative % |  |
| --- | --- | --- | --- |
| 1. | 0,01221081 | 71,025 | 71,025 |
| 2. | 0,00203360 | 11,829 | 82,853 |
| 3. | 0,00055644 | 3,237 | 86,090 |
| 4. | 0,00046761 | 2,720 | 88,810 |
| 5. | 0,00033573 | 1,953 | 90,763 |
| 6. | 0,00024623 | 1,432 | 92,195 |
| 7. | 0,00022920 | 1,333 | 93,528 |
| 8. | 0,00017939 | 1,043 | 94,571 |
| 9. | 0,00015712 | 0,914 | 95,485 |
| 10. | 0,00011565 | 0,673 | 96,158 |
| 11. | 0,00009825 | 0,571 | 96,729 |
| 12. | 0,00007449 | 0,433 | 97,163 |
| 13. | 0,00006712 | 0,390 | 97,553 |
| 14. | 0,00004711 | 0,274 | 97,827 |
| 15. | 0,00004144 | 0,241 | 98,068 |
| 16. | 0,00003693 | 0,215 | 98,283 |
| 17. | 0,00003244 | 0,189 | 98,472 |
| 18. | 0,00002776 | 0,161 | 98,633 |
| 19. | 0,00002483 | 0,144 | 98,778 |
| 20. | 0,00002109 | 0,123 | 98,900 |
| 21. | 0,00001902 | 0,111 | 99,011 |
| 22. | 0,00001880 | 0,109 | 99,120 |
| 23. | 0,00001654 | 0,096 | 99,216 |
| 24. | 0,00001332 | 0,077 | 99,294 |
| 25. | 0,00001279 | 0,074 | 99,368 |
| 26. | 0,00000997 | 0,058 | 99,426 |
| 27. | 0,00000971 | 0,056 | 99,483 |
| 28. | 0,00000836 | 0,049 | 99,531 |
| 29. | 0,00000823 | 0,048 | 99,579 |
| 30. | 0,00000710 | 0,041 | 99,621 |
| 31. | 0,00000621 | 0,036 | 99,657 |
| 32. | 0,00000605 | 0,035 | 99,692 |
| 33. | 0,00000520 | 0,030 | 99,722 |
| 34. | 0,00000477 | 0,028 | 99,750 |
| 35. | 0,00000414 | 0,024 | 99,774 |
| 36. | 0,00000404 | 0,024 | 99,797 |
| 37. | 0,00000380 | 0,022 | 99,819 |
| 38. | 0,00000317 | 0,018 | 99,838 |
| 39. | 0,00000286 | 0,017 | 99,855 |
| 40. | 0,00000264 | 0,015 | 99,870 |
| 41. | 0,00000250 | 0,015 | 99,884 |
| 42. | 0,00000239 | 0,014 | 99,898 |
| 43. | 0,00000218 | 0,013 | 99,911 |
| 44. | 0,00000178 | 0,010 | 99,921 |
| 45. | 0,00000171 | 0,010 | 99,931 |
| 46. | 0,00000147 | 0,009 | 99,940 |
| 47. | 0,00000131 | 0,008 | 99,948 |
| 48. | 0,00000115 | 0,007 | 99,954 |
| 49. | 0,00000112 | 0,006 | 99,961 |
| 50. | 0,00000107 | 0,006 | 99,967 |
| 51. | 0,00000101 | 0,006 | 99,973 |
| 52. | 0,00000079 | 0,005 | 99,977 |
| 53. | 0,00000077 | 0,004 | 99,982 |
| 54. | 0,00000064 | 0,004 | 99,986 |
| 55. | 0,00000060 | 0,003 | 99,989 |
| 56. | 0,00000053 | 0,003 | 99,992 |
| 57. | 0,00000043 | 0,003 | 99,995 |
| 58. | 0,00000037 | 0,002 | 99,997 |
| 59. | 0,00000028 | 0,002 | 99,999 |
| 60. | 0,00000026 | 0,001 | 100,000 |
| Total variance: 0,01719233 | | | |
| Variance of the eigenvalues: 0,0000024861244 | | | |
| Eigenvalue variance scaled by total variance: 0,00841 | | | |
| Eigenvalue variance scaled by total variance and number of variables: 0,51322 | | | |

Supp. Table 3. General PCA Eigenvalues

**Tab. 4. PCA Scores: PC1 and PC2** (Fig. 5)

| Id | Position | PC1 | PC2 |
| --- | --- | --- | --- |
| Ind6.F.1 | 1 | -0,09053 | 0,017375 |
| Ind5.F.1 | 1 | -0,10226 | -0,04034 |
| Ind14.F.1 | 1 | -0,09623 | 0,084354 |
| Ind7.M.1 | 1 | -0,10499 | -0,06238 |
| Ind9.F.1 | 1 | -0,06993 | -0,13426 |
| Ind10.F.1 | 1 | -0,02784 | -0,136 |
| Ind11.M.1 | 1 | -0,15389 | 0,004355 |
| Ind12.M.1 | 1 | -0,02195 | -0,08116 |
| Ind1.F.1 | 1 | -0,13797 | -0,02639 |
| Ind2.F.1 | 1 | -0,13931 | 0,076069 |
| Ind3.F.1 | 1 | -0,16002 | 0,024745 |
| Ind4.F.1 | 1 | -0,16014 | 0,031268 |
| Ind13.F.1 | 1 | -0,08785 | 0,017055 |
| Ind20.M.1 | 1 | -0,1235 | 0,020095 |
| Ind15.M.1 | 1 | -0,03909 | 0,082342 |
| Ind16.F.1 | 1 | -0,08524 | 0,041046 |
| Ind19.F.1 | 1 | -0,04571 | -0,03382 |
| Ind18.M.1 | 1 | -0,09528 | -0,00929 |
| Ind22.F.1 | 1 | -0,05426 | -0,01735 |
| Ind25.F.1 | 1 | -0,11576 | 0,073508 |
| Ind26.F.1 | 1 | -0,1011 | 0,026674 |
| Ind27.F.1 | 1 | -0,07458 | -0,02434 |
| Ind29.F.1 | 1 | -0,09054 | -0,0478 |
| Ind8.M.1 | 1 | -0,04921 | 0,032591 |
| Ind24.M.1 | 1 | -0,14723 | 0,015579 |
| Ind30.F.1 | 1 | -0,06391 | 0,0457 |
| Ind31.M.1 | 1 | -0,13031 | -0,01139 |
| Ind32.M.1 | 1 | -0,15284 | -0,04552 |
| Ind33.M.1 | 1 | -0,05561 | 0,013036 |
| Ind34.F.1 | 1 | -0,09876 | -0,03864 |
| Ind35.F.1 | 1 | -0,1232 | -0,01472 |
| Ind36.M.1 | 1 | -0,06341 | -0,05789 |
| Ind37.F.1 | 1 | -0,03126 | -0,07656 |
| Ind38.M.1 | 1 | -0,12568 | -0,01669 |
| Ind39.M.1 | 1 | -0,16616 | -0,0117 |
| Ind40.F.1 | 1 | -0,06671 | 0,035781 |
| Ind41.F.1 | 1 | -0,16346 | 0,030458 |
| Ind42.M.1 | 1 | -0,166 | 0,013016 |
| Ind44.F.1 | 1 | -0,10982 | -0,07368 |
| Ind45.F.1 | 1 | -0,19457 | 0,05169 |
| Ind46.M.1 | 1 | -0,14063 | -0,109 |
| Ind47.F.1 | 1 | -0,15717 | -0,01681 |
| Ind48.F.1 | 1 | -0,08606 | -0,07408 |
| Ind49.F.1 | 1 | -0,11814 | -0,00345 |
| Ind50.M.1 | 1 | -0,12114 | -0,03841 |
| Ind57.F.1 | 1 | -0,05243 | -0,0336 |
| Ind59.M.1 | 1 | -0,07183 | -0,02777 |
| Ind58.F.1 | 1 | -0,02786 | -0,01324 |
| Ind53.M.1 | 1 | -0,16407 | 0,011389 |
| Ind52.F.1 | 1 | -0,11199 | 0,013867 |
| Ind51.M.1 | 1 | -0,17189 | 0,003255 |
| Ind54.F.1 | 1 | -0,16908 | -0,03744 |
| Ind55.M.1 | 1 | -0,15831 | -0,00947 |
| Ind56.F.1 | 1 | -0,13645 | 0,009898 |
| Ind60.M.1 | 1 | -0,17204 | 0,0045 |
| Ind62.F.1 | 1 | -0,13178 | -0,00529 |
| Ind61.F.1 | 1 | -0,16962 | -0,00408 |
| Ind64.M.1 | 1 | -0,18631 | -0,02742 |
| Ind63.M.1 | 1 | -0,1043 | -0,08911 |
| Ind65.M.1 | 1 | -0,12926 | -0,12839 |
| Ind67.M.1 | 1 | -0,21566 | -0,01613 |
| Ind68.M.1 | 1 | -0,23184 | -0,01568 |
| Ind69.M.1 | 1 | -0,15572 | -0,00794 |
| Ind70.M.1 | 1 | -0,18304 | 0,04714 |
| Ind71.M.1 | 1 | -0,08502 | -0,03143 |
| Ind72.M.1 | 1 | -0,04204 | -0,01113 |
| Ind73.M.1 | 1 | -0,09846 | 0,016575 |
| Ind74.M.1 | 1 | -0,18043 | 0,010051 |
| Ind75.M.1 | 1 | -0,05201 | 0,0112 |
| Ind66.M.1 | 1 | -0,20388 | -0,03995 |
| Ind6.F.2 | 2 | -0,02098 | 0,040323 |
| Ind5.F.2 | 2 | -0,04669 | -0,01832 |
| Ind14.F.2 | 2 | 0,002642 | 0,048887 |
| Ind8.M.2 | 2 | 0,089876 | -0,00243 |
| Ind9.F.2 | 2 | -0,01828 | -0,00696 |
| Ind10.F.2 | 2 | 0,045483 | -0,04865 |
| Ind11.M.2 | 2 | -0,03951 | 0,003753 |
| Ind12.M.2 | 2 | 0,067737 | -0,07915 |
| Ind1.F.2 | 2 | -0,01594 | 0,053575 |
| Ind2.F.2 | 2 | -0,03275 | 0,038984 |
| Ind3.F.2 | 2 | -0,03872 | 0,05367 |
| Ind4.F.2 | 2 | -0,04893 | 0,053662 |
| Ind13.F.2 | 2 | -0,02315 | 0,044818 |
| Ind20.M.2 | 2 | -0,01295 | 0,084853 |
| Ind15.M.2 | 2 | 0,042166 | 0,079336 |
| Ind16.F.2 | 2 | 0,004443 | 0,065011 |
| Ind19.F.2 | 2 | 0,068674 | 0,024177 |
| Ind18.M.2 | 2 | 0,037217 | 0,005161 |
| Ind22.F.2 | 2 | 0,080532 | 0,005418 |
| Ind25.F.2 | 2 | -0,02659 | 0,06254 |
| Ind26.F.2 | 2 | -0,02697 | 0,020032 |
| Ind27.F.2 | 2 | 0,038936 | -0,00761 |
| Ind29.F.2 | 2 | -0,00583 | -0,0185 |
| Ind7.M.2 | 2 | 0,054899 | -0,01377 |
| Ind24.M.2 | 2 | -0,10498 | -0,00626 |
| Ind30.F.2 | 2 | -0,06792 | 0,035946 |
| Ind31.M.2 | 2 | -0,04851 | -0,00763 |
| Ind32.M.2 | 2 | -0,03953 | -0,0105 |
| Ind33.M.2 | 2 | 0,054904 | 0,017997 |
| Ind34.F.2 | 2 | 0,023233 | -0,02072 |
| Ind35.F.2 | 2 | -0,03955 | 0,034473 |
| Ind36.M.2 | 2 | 0,06526 | -0,00766 |
| Ind37.F.2 | 2 | 0,043371 | -0,05386 |
| Ind38.M.2 | 2 | -0,03318 | 0,010142 |
| Ind39.M.2 | 2 | 0,0162 | 0,038624 |
| Ind40.F.2 | 2 | -0,06294 | 0,044015 |
| Ind41.F.2 | 2 | -0,01393 | 0,090663 |
| Ind42.M.2 | 2 | -0,03937 | 0,079628 |
| Ind44.F.2 | 2 | -0,00684 | -0,00926 |
| Ind45.F.2 | 2 | -0,04273 | 0,03856 |
| Ind46.M.2 | 2 | 0,027658 | 0,002911 |
| Ind47.F.2 | 2 | -0,00941 | 0,049452 |
| Ind48.F.2 | 2 | 0,027368 | -0,01585 |
| Ind49.F.2 | 2 | -0,05093 | 0,014722 |
| Ind50.M.2 | 2 | -0,01744 | -0,02182 |
| Ind59.M.2 | 2 | 0,03058 | -0,0338 |
| Ind58.F.2 | 2 | -0,00498 | 0,013294 |
| Ind57.F.2 | 2 | 0,030661 | 0,040981 |
| Ind53.M.2 | 2 | -0,11381 | -0,02556 |
| Ind52.F.2 | 2 | 0,015909 | -0,01994 |
| Ind51.M.2 | 2 | -0,06488 | -0,03625 |
| Ind54.F.2 | 2 | -0,07565 | -0,00577 |
| Ind55.M.2 | 2 | -0,07475 | 0,046128 |
| Ind56.F.2 | 2 | -0,06213 | 0,023402 |
| Ind60.M.2 | 2 | -0,02541 | -0,01242 |
| Ind61.F.2 | 2 | -0,03446 | -0,01229 |
| Ind64.M.2 | 2 | -0,0274 | -0,04006 |
| Ind62.F.2 | 2 | 0,022571 | -0,00961 |
| Ind63.M.2 | 2 | 0,005337 | -0,06134 |
| Ind65.M.2 | 2 | -0,0058 | -0,11765 |
| Ind67.M.2 | 2 | -0,10069 | -0,02054 |
| INd68.M.2 | 2 | -0,05144 | 0,049282 |
| Ind69.M.2 | 2 | -0,08142 | 0,010881 |
| Ind66.M.2 | 2 | -0,01744 | -0,03495 |
| Ind70.M.2 | 2 | -0,09829 | 0,062468 |
| Ind71.M.2 | 2 | -0,01079 | 0,032422 |
| Ind72.M.2 | 2 | 0,057642 | 0,043308 |
| Ind73.M.2 | 2 | 0,062028 | 0,009124 |
| Ind74.M.2 | 2 | -0,08711 | 0,017976 |
| Ind75.M.2 | 2 | 0,020715 | 0,046617 |
| Ind14.F.3 | 3 | 0,11326 | 0,043669 |
| Ind6.F.3 | 3 | 0,086105 | 0,027307 |
| Ind5.F.3 | 3 | 0,070213 | -0,00286 |
| Ind7.M.3 | 3 | 0,118542 | 0,005275 |
| Ind8.M.3 | 3 | 0,132822 | -0,00652 |
| Ind9.F.3 | 3 | 0,112558 | 0,043914 |
| Ind10.F.3 | 3 | 0,158557 | -0,06519 |
| Ind11.M.3 | 3 | 0,10493 | 0,005516 |
| Ind12.M.3 | 3 | 0,124361 | -0,07851 |
| Ind4.F.3 | 3 | 0,123938 | 0,039074 |
| Ind1.F.3 | 3 | 0,114731 | 0,051517 |
| Ind2.F.3 | 3 | 0,168179 | 0,009612 |
| Ind3.F.3 | 3 | 0,134744 | -0,02916 |
| Ind13.F.3 | 3 | 0,104454 | 0,009876 |
| Ind20.M.3 | 3 | 0,155572 | 0,061309 |
| Ind15.M.3 | 3 | 0,132617 | 0,129611 |
| Ind16.F.3 | 3 | 0,101499 | 0,043104 |
| Ind19.F.3 | 3 | 0,18459 | 0,040441 |
| Ind18.M.3 | 3 | 0,139157 | 0,036315 |
| Ind22.F.3 | 3 | 0,202282 | -0,02871 |
| Ind25.F.3 | 3 | 0,161606 | -5,19E-04 |
| Ind26.F.3 | 3 | 0,161973 | -0,02517 |
| Ind.27.F3 | 3 | 0,151833 | -0,06282 |
| Ind29.F.3 | 3 | 0,115822 | -0,07604 |
| Ind24.M.3 | 3 | 0,023999 | 0,037946 |
| Ind30.F.3 | 3 | 0,131241 | 0,062082 |
| Ind31.M.3 | 3 | 0,169165 | 0,0017 |
| Ind32.M.3 | 3 | 0,118247 | -0,03524 |
| Ind33.M.3 | 3 | 0,16835 | -0,05668 |
| Ind34.F.3 | 3 | 0,158146 | -0,05061 |
| Ind35.F.3 | 3 | 0,065872 | 0,050908 |
| Ind36.M.3 | 3 | 0,141544 | -0,00852 |
| Ind37.F.3 | 3 | 0,12156 | -0,06055 |
| Ind38.M.3 | 3 | 0,080575 | 0,012756 |
| Ind39.M.3 | 3 | 0,110753 | 0,002073 |
| Ind40.F.3 | 3 | 0,133348 | 0,062293 |
| Ind42.M.3 | 3 | 0,094036 | 0,030432 |
| Ind41.F.3 | 3 | 0,129285 | 0,036988 |
| Ind44.F.3 | 3 | 0,120731 | 0,012496 |
| Ind45.F.3 | 3 | 0,10419 | 0,053307 |
| Ind46.M.3 | 3 | 0,16807 | -0,08888 |
| Ind47.F.3 | 3 | 0,114909 | 0,009195 |
| Ind48.F.3 | 3 | 0,186031 | -0,01132 |
| Ind49.F.3 | 3 | 0,195931 | -0,00487 |
| Ind50.M.3 | 3 | 0,162024 | -0,0036 |
| Ind58.F.3 | 3 | 0,07205 | 0,04986 |
| Ind59.M.3 | 3 | 0,165441 | -0,05925 |
| Ind57.F.3 | 3 | 0,135673 | 0,027824 |
| Ind53.M.3 | 3 | 0,043749 | 0,017739 |
| Ind52.F.3 | 3 | 0,166152 | -0,04041 |
| Ind51.M.3 | 3 | 0,076277 | -0,0152 |
| Ind54.F.3 | 3 | 0,099776 | 0,036232 |
| Ind55.M.3 | 3 | 0,024995 | 0,020352 |
| Ind56.F.3 | 3 | 0,13986 | -0,03622 |
| Ind60.M.3 | 3 | 0,124827 | 0,035027 |
| Ind63.M.3 | 3 | 0,114599 | 0,018843 |
| Ind61.F.3 | 3 | 0,187439 | -0,06764 |
| Ind64.M.3 | 3 | 0,131224 | -0,04703 |
| Ind62.F.3 | 3 | 0,176978 | -0,01626 |
| Ind65.M.3 | 3 | 0,131484 | -0,122 |
| Ind67.M.3 | 3 | 0,078181 | -0,00676 |
| Ind68.M.3 | 3 | 0,079294 | 0,041 |
| Ind69.M.3 | 3 | 0,186228 | -0,04116 |
| Ind66.M.3 | 3 | 0,088248 | -0,01012 |
| Ind70.M.3 | 3 | 0,051545 | 0,055017 |
| Ind71.M.3 | 3 | 0,149013 | -0,00832 |
| Ind72.M.3 | 3 | 0,20621 | -2,45E-04 |
| Ind73.M.3 | 3 | 0,149012 | -0,04847 |
| Ind74.M.3 | 3 | 0,193636 | 0,035649 |
| Ind75.M.3 | 3 | 0,10526 | 0,03564 |

Supp Table 4: PC Scores of PC1 and PC2 for each individual

**Tab. 5 and 6. PC Scores tests between groups:**

PC1:

Kruskal-Wallis: p (same): 6,655E-38*

Dunn´s post hoc:

|  | Position 1 -PC1 | Position 2 - PC1 | Position 3 - PC1 |
| --- | --- | --- | --- |
| Position 1 -PC1 |  | 2,041E-09* | 4,907E-39* |
| Position 2 - PC1 | 2,041E-09* |  | 1,493E-12* |
| Position 3 - PC1 | 4,907E-39* | 1,493E-12* |  |

Supp. Table 5. PC1 scores analysis

PC2:

Kruskal-Wallis: PC2: p (same): 0,01426*

Dunn´s post hoc:

|  | Position 1 -PC2 | Position 2- PC2 | Position 3- PC2 |
| --- | --- | --- | --- |
| Position 1 -PC2 |  | 0,003733* | 0,08713 |
| Position 2- PC2 | 0,003733* |  | 0,2344 |
| Position 3- PC2 | 0,08713 | 0,2344 |  |

Supp. Table 6. PC2 scores analysis

**Tab.7. Regression results:**

| Sums of squares | |
| --- | --- |
| Total SS: | 3,59319698 |
| Predicted SS: | 2,08855415 |
| Residual SS: | 1,50464284 |
| % predicted: | 58,1252% |
| Permutation test against the null hypothesis of independence  Number of randomization rounds: 100 | |
| P-value: | <.01 |

*Supp. Table 7. Regression results*

**Tab. 8. Principal Component Analysis. Regression residuals** (Fig, 6):

|  | Eigenvalues | % Variance | Cumulative % |
| --- | --- | --- | --- |
| 1 | 0,00271213 | 37,672 | 37,672 |
| 2 | 0,00163038 | 22,646 | 60,319 |
| 3 | 0,00051401 | 7,140 | 67,459 |
| 4 | 0,00045164 | 6,273 | 73,732 |
| 5 | 0,00032838 | 4,561 | 78,294 |
| 6 | 0,00024069 | 3,343 | 81,637 |
| 7 | 0,00022718 | 3,156 | 84,792 |
| 8 | 0,00017712 | 2,460 | 87,253 |
| 9 | 0,00015686 | 2,179 | 89,431 |
| 10 | 0,00011272 | 1,566 | 90,997 |
| 11 | 0,00009321 | 1,295 | 92,292 |
| 12 | 0,00007361 | 1,022 | 93,314 |
| 13 | 0,00006488 | 0,901 | 94,215 |
| 14 | 0,00004693 | 0,652 | 94,867 |
| 15 | 0,00004134 | 0,574 | 95,441 |
| 16 | 0,00003581 | 0,497 | 95,939 |
| 17 | 0,00003236 | 0,450 | 96,388 |
| 18 | 0,00002742 | 0,381 | 96,769 |
| 19 | 0,00002479 | 0,344 | 97,114 |
| 20 | 0,00002039 | 0,283 | 97,397 |
| 21 | 0,00001899 | 0,264 | 97,661 |
| 22 | 0,00001859 | 0,258 | 97,919 |
| 23 | 0,00001589 | 0,221 | 98,140 |
| 24 | 0,00001324 | 0,184 | 98,324 |
| 25 | 0,00001274 | 0,177 | 98,500 |
| 26 | 0,00000996 | 0,138 | 98,639 |
| 27 | 0,00000963 | 0,134 | 98,773 |
| 28 | 0,00000830 | 0,115 | 98,888 |
| 29 | 0,00000818 | 0,114 | 99,002 |
| 30 | 0,00000688 | 0,096 | 99,097 |
| 31 | 0,00000620 | 0,086 | 99,183 |
| 32 | 0,00000605 | 0,084 | 99,267 |
| 33 | 0,00000520 | 0,072 | 99,339 |
| 34 | 0,00000477 | 0,066 | 99,406 |
| 35 | 0,00000413 | 0,057 | 99,463 |
| 36 | 0,00000403 | 0,056 | 99,519 |
| 37 | 0,00000377 | 0,052 | 99,572 |
| 38 | 0,00000315 | 0,044 | 99,615 |
| 39 | 0,00000286 | 0,040 | 99,655 |
| 40 | 0,00000263 | 0,037 | 99,691 |
| 41 | 0,00000249 | 0,035 | 99,726 |
| 42 | 0,00000238 | 0,033 | 99,759 |
| 43 | 0,00000217 | 0,030 | 99,789 |
| 44 | 0,00000177 | 0,025 | 99,814 |
| 45 | 0,00000170 | 0,024 | 99,838 |
| 46 | 0,00000143 | 0,020 | 99,857 |
| 47 | 0,00000130 | 0,018 | 99,875 |
| 48 | 0,00000115 | 0,016 | 99,891 |
| 49 | 0,00000111 | 0,015 | 99,907 |
| 50 | 0,00000107 | 0,015 | 99,922 |
| 51 | 0,00000098 | 0,014 | 99,935 |
| 52 | 0,00000079 | 0,011 | 99,946 |
| 53 | 0,00000076 | 0,010 | 99,957 |
| 54 | 0,00000064 | 0,009 | 99,966 |
| 55 | 0,00000060 | 0,008 | 99,974 |
| 56 | 0,00000052 | 0,007 | 99,981 |
| 57 | 0,00000043 | 0,006 | 99,987 |
| 58 | 0,00000037 | 0,005 | 99,993 |
| 59 | 0,00000028 | 0,004 | 99,996 |
| 60 | 0,00000026 | 0,004 | 100,000 |
| Total variance: 0,00719925 | | | |
| Variance of the eigenvalues: 0,0000001655411 | | | |
| Eigenvalue variance scaled by total variance: 0,00319 | | | |
| Eigenvalue variance scaled by total variance and number of variables: 0,19489 | | | |

*Supp. Table 8. Residuals PCA results*
